# Exploring hepatitis E virus seroprevalence and associated risk factors among the human population in Tandil, Buenos Aires, Argentina

**DOI:** 10.3389/fpubh.2023.1257754

**Published:** 2023-10-05

**Authors:** Mariana Alejandra Rivero, Lorena Paola Arce, Silvina Elena Gutiérrez, Adela Tisnés, Juan Antonio Passucci, Julia Analia Silva, Ayelén Barón Prato, Florencia Sánchez, Julia Matias Brancher, Silvia Marcela Estein, María Guadalupe Vizoso-Pinto

**Affiliations:** ^1^Centro de Investigación Veterinaria de Tandil (UNCPBA-CICPBA-CONICET), Facultad de Ciencias Veterinarias, Universidad Nacional del Centro de la Provincia de Buenos Aires (UNCPBA), Tandil, Argentina; ^2^Laboratorio de Biología de las Infecciones, Instituto Superior de Investigaciones Biológicas (INSIBIO), CONICET-UNT, Tucumán, Argentina; ^3^Laboratorio de Ciencias Básicas Or. Genética, Facultad de Medicina de la Universidad Nacional de Tucumán, Tucumán, Argentina; ^4^Facultad de Ciencias Humanas, CIG- IGEHCS- CONICET, Universidad Nacional del Centro de la Provincia de Buenos Aires (UNCPBA), Tandil, Argentina

**Keywords:** hepatitis E virus (HEV), seroprevalence, risk factors, spatial analyses, water exposure

## Abstract

**Background:**

Hepatitis E virus (HEV) infection is a common cause of acute clinical hepatitis worldwide and is emerging as a disease in Argentina. It is primarily transmitted through contaminated water and food, following the fecal-oral route. Furthermore, is a zoonotic disease with swine as the primary reservoir. Prevalence of HEV infection in humans in several regions of Argentina remains unknown.

**Objectives:**

(i) Determine the seroprevalence of HEV among the human population in Tandil, Buenos Aires, Argentina; (ii) Evaluate its association with demographic, socioeconomic and other risk exposures variables, and (iii) Describe and analyze spatial patterns related to HEV infection.

**Methods:**

From August 2020 to July 2021, serum samples were collected from 969 individuals aged 1–80 years. Seroprevalence and 95% Confidence Interval was determined. To assess the factors associated with the presence of anti-HEV antibodies, associations between the variables and seropositivity were evaluated through bivariate and multivariate analysis. Spatial scanning for clusters of positivity was carried out. Factors associated with these clusters were also assessed.

**Results:**

Anti-HEV antibodies were detected in 4.64% (IC 95% 3.27–6.02) of samples. Dark urine was associated with seropositivity (*p* = 0.02). Seropositivity was linked with the presence of natural water courses near their households (*p* = 0.02); the age (*p* = 0.04); and previous travel to Europe (*p* = 0.04). A spatial cluster of low rates of HEV seropositivity was detected, with greater distance of the households to water courses associated to the cluster, and male sex inversely associated to it.

**Discussion and conclusion:**

This study is the first study to investigate the prevalence of HEV in the population from Tandil, Buenos Aires, Argentina. Considering HEV infection in the differential diagnosis in individuals presenting acute hepatitis is highlighted. The incorporation of HEV testing into blood screening policies should be mandatory. Factors related to the infection and spatial patterns of high and low risk were determined, and should be considered when implementing specific preventive measures.

## Introduction

Hepatitis E virus (HEV) infection is a worldwide emerging disease and a public health concern. It is also the first cause of acute viral hepatitis in the world with a disease burden of 20 million HEV infections worldwide, 3.3 million symptomatic cases and 56,000 deaths every year (1, 2). As new HEV subtypes have been identified from animal, human, and environmental isolates, new potential animal reservoirs have emerged, and evidence on the zoonotic transmission of the virus from animal hosts and the environment was provided (3). HEV genotypes 1 and 2 are restricted to humans and the infection is caused by accidental fecal contamination of drinking water. HEV genotypes 3, 4 and 7 are primarily zoonotic; interspecies transmission occurs through direct contact with infected animals and consumption of undercooked HEV-contaminated food. Parenteral transmission via transfusion of blood products has also been described (1, 2, 4, 5).

The progression of acute hepatitis E is often mild with spontaneous resolution being the normal. Interestingly, over 60% of infections display no symptoms at all. When symptoms are present, they often resemble those observed in acute hepatitis A, with approximately 65% of symptomatic cases exhibiting jaundice. Typical symptoms include asthenia, diarrhea, nausea, and/or vomiting, abdominal pain, fever, arthralgia, dark urine and light (clay/ash-colored) stool are common. Pruritus and/or upper right quadrant pain may also be present. Extrahepatic manifestations such as hematological, neurological, and renal disorders have also been described. In some cases, it can result in severe acute hepatitis. HEV infection in immunocompromised patients can cause chronic hepatitis leading to cirrhosis, and fulminant hepatitis. Pregnant women are more susceptible to fulminant hepatitis and obstetric complications, mainly during the third quarter of pregnancy. The mortality rate for adults in an epidemic area is 0.2–4.0%. However, in patients with chronic liver disease and pregnant women, the mortality rates can be significantly higher, reaching up to 70 and 25%, respectively (1, 2, 6, 7).

There is poor awareness of the disease among physicians. Thus, routine check for the disease is rarely conducted in the hospitals in most parts of the world. Thereby leading to misdiagnosis and underdiagnosis of the disease (5).

Seroprevalence rates of HEV among blood donors vary across continents. In Europe, some countries have reported relatively high seroprevalence. Bulgaria conducted a study with a prevalence of 25.9%, while Croatia and Serbia reported rates of 21.5 and 15.0%, respectively. France has shown a broad seroprevalence ranging from 3.2% to as high as 52.0% among different blood donor populations. Denmark, England, Greece, Italy, Spain, Germany, and Switzerland have reported seroprevalence rates of 20.6, 16.0, 9.43, 8.7, 7.3, 6.8, and 4.9%, respectively, with a significant number of infections acquired locally. Moving to South Africa, Thailand, and India in the African and Asian continents, seroprevalences have been notably higher, with values of 42.8, 29.7, and 17.7%, respectively. In contrast, in Japan seroprevalence reported is very low (3.4%) (5, 6).

The seroprevalence of HEV within different population groups and regions of the Americas can range from 0 to 40.6%, as reported by Fernandez Villalobos et al. (2). The USA and Canada had seroprevalences of 18.3 and 5.9% (5, 6). In South America, studies conducted among blood donors have shown current prevalence rates ranging from 1.8 to 9.8%, indicating moderate circulation of HEV in the region. However, there is limited research on the overall epidemiology of HEV in South America, and the burden of the disease remains largely unknown. First serological studies in the continent were conducted during the 1990s and early 2000s, revealing prevalence rates ranging from 0.1 to 8% among both rural and urban populations. In 2011, studies conducted in Bolivia, Brazil, and Colombia reported varying seroprevalences of HEV (8–12).

In a previous study performed in a non-endemic area from Argentina, the seroprevalence was very low (1.80%) (13). In other studies, in the northwest region of the country, the HEV seroprevalences were 5.6% (95% CI: 2.3–11.2%) for an indigenous population, 9.23% for blood donors in Tucumán Province (Argentina) and 9% in Salta province (14–16). In the central region, Córdoba city (Argentina), the overall IgG anti-HEV prevalence obtained in blood donors (*n* = 547) was 3.47% (17). Di Lello et al. (18) conducted a study in blood donors (*n* = 391) of five Argentinian regions and found that HEV seropositivity varied from 5.1 to 20.0%.

There is a lack of systematically retrieved evidence on the seroprevalence and risk factors of HEV in Argentina in general and in Tandil (Buenos Aires province) in particular, which is a medium-sized city located in the central region of Argentina, where farming is one of the main economic activities. Therefore, and in order to provide evidence for targeted prevention strategies we carried out this study from a random sample that allowed an inference to be made to the entire population and was also carried out under the One Health approach (4). To address this research gap and provide valuable insights for targeted prevention strategies, this study aimed to achieve the following objectives: (i) Determine the seroprevalence of HEV among the human population in Tandil, Buenos Aires, Argentina. (ii) Evaluate the association between HEV seroprevalence and various demographic, socioeconomic, and other risk exposure variables. (iii) Describe and analyze the spatial patterns associated with HEV infection.

## Materials and methods

### Study area and population

**Figure 1:**
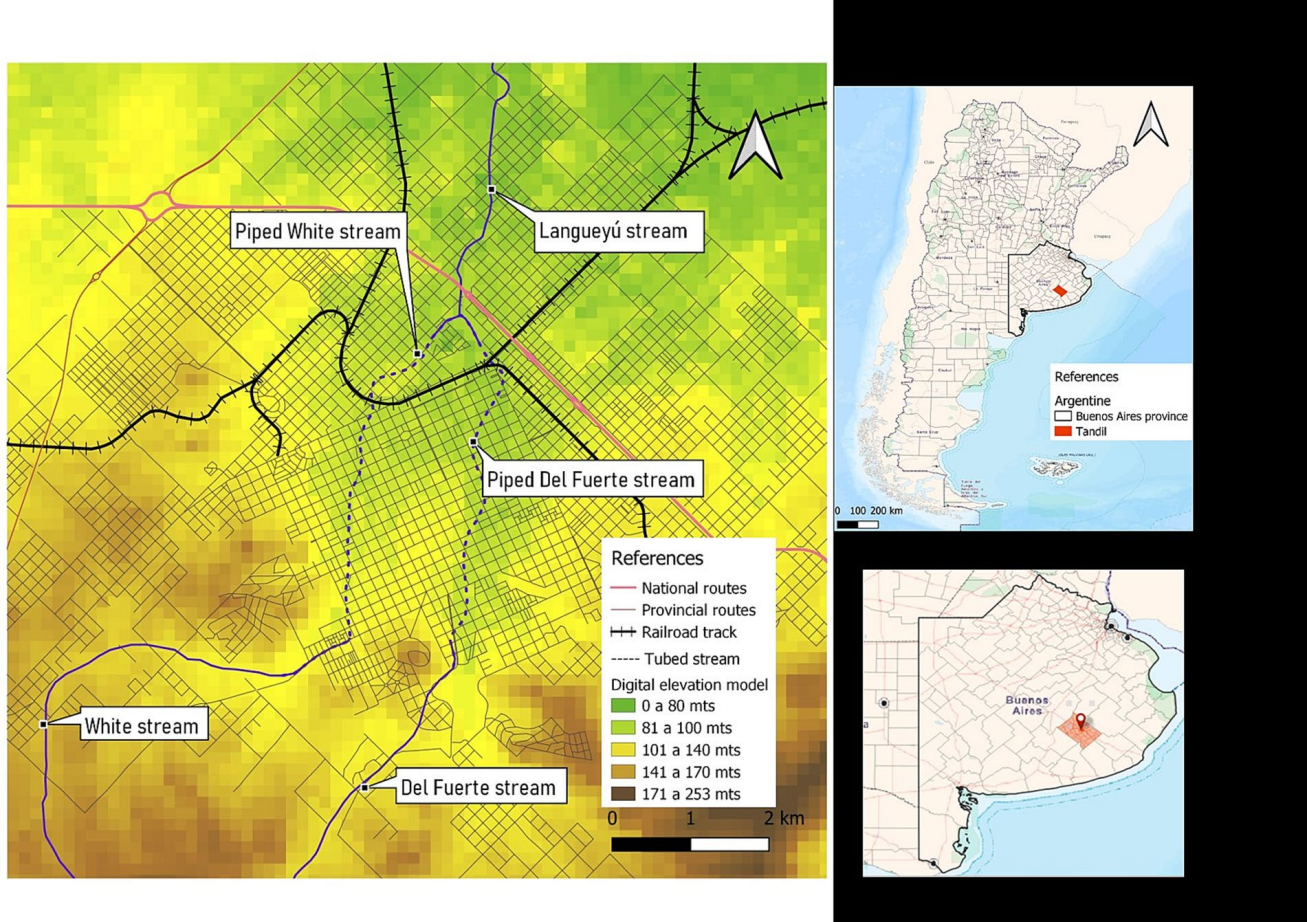
Map showing the location of the city of Tandil, in the province of Buenos Aires, Argentina. Digital elevation model and water courses.

The study area comprises the South-eastern region of Buenos Aires province (Argentina), in which it is located the Tandilia mountain system. This region is characterized by agricultural and livestock production and sustains the biggest industrial concentration of the country and the most important urban settlements. Climatic regime along the study area is subhumid to humid mesothermal, with little or no water deficiency during summer months (December, January, and February). Over the course of the year, the absolute maximum temperatures range 37–39°C, and the absolute minimum range −6 to −7°C (19). The present study was carried out specifically in Tandil city, the head town of the Tandil district (coordinates 37°19′00″S; 59°08′00″W; area 52,34 km^2^). This is a community located approximately 360 km from Buenos Aires capital city, with a total population of 150,162 inhabitants (20). In Figure 1, the map displays the location of the city on a chain of valleys that descend from the Tandilia mountain system, to the South and West, a transition to the mountain foothills, located to the North and Northeast, with smoother and the plain area, slopes to the north of the urban area. The most important basin is that of the Langueyú System, which crosses the entire urban area of the city. The Langueyú stream originates from the merging of Del Fuerte and White surface courses, which were enclosed in tubes during the 70’s and 80’s. The study area encompasses both urban and peri-urban regions, with the latter being near agricultural and livestock activities. The process of urban expansion has occurred along two axes of expansion: one of them to the Northwest, and another to the North Center East and South East. The first axis is notable for its susceptibility to flooding during precipitation events, and it is located nearby the Langueyú stream (21) (Figure 1). Tandil city possesses a water supply network coverage of approximately 90%, but only a 60% coverage of sewage network. The sewage network is only for effluents, because the rest of the urban drainage is channeled through a stormwater drainage network. Although the sewage network and sewage treatment plants are adequate, their operation is altered by clandestine connections of household stormwater drains to the sewage network. Furthermore, the stormwater drainage network, which includes piped streams, is affected by clandestine connections of industrial and sewage effluents (19).

### Sample collection and detection of IgG antibodies against HEV

Between August 2020 and July 2021, serum samples were collected from 969 individuals aged 1–80 years (median 44) by a random spatial sampling method. To assemble the sample of people to be analyzed, an anonymous list of all the people included in the digital medical records of the Tandil Public Health Integrated System was created. This list accounted for about 95% of the city’s total population and formed the sampling frame. A spatial random sampling without replacement was applied, considering the unequal distribution of the population according to age. The calculated minimum sample size was 783 assuming a seroprevalence of 9% with an absolute error of 2% and a confidence level of 95%. Immunocompromised individuals were excluded by asking about the history of immunosuppressive diseases or medications. Anti-HEV IgG was detected by a validated indirect in-house ELISA as described in Arce et al. (14) [sensitivity (93.33%), specificity (99.4%), and agreement (κ index = 0.94)], and seroprevalence within a 95% Confidence Interval (CI) was determined.

### Data collection

To assess the factors associated with the presence of anti-HEV antibodies, those individuals who agreed to be surveyed were interviewed by a trained interviewer using a semi-structured questionnaire designed *ad hoc* to obtain individual information on clinical and epidemiological data (Appendix 1). Information from these questionnaires included: socio-demographic, and housing characteristics, behaviors, education, occupation, animal and environmental exposure and clinical data. Data about knowledge of HEV infection and prevention measures were also collected. All the households of participants were geo-referenced using Global Positioning Systems (GPS).

### Statistical analysis

Data analysis included descriptive statistics of means (with standard deviation) and medians (with first and third quartiles), depending on the distribution of the data, while categorical variables were expressed as percentages (%). The association between outcome (positivity) and the variables under analysis were assessed in two-way contingency table analyses using Pearson’s Chi-Squared Test or Fisher’s exact test if the expected value of one or more cells was less than 5. For quantitative variables two-sided Student’s *t* Test or Wilcoxon’s rank sum test were used. The null hypothesis stated that there were no differences between groups. Factors having significant *p* < 0.20 were selected and included in a multivariate logistic regression model. The maximum likelihood with a convergence criterion of 0.01 for a maximum of 10 interactions was used as the estimation method. The significance level was *p* < 0.05. The strength of association between each co-variable and seroprevalence was calculated and expressed as an estimated value by the adjusted OR and their respective 95% CI. To identify possible confusion factors, association between variables was assessed by χ^2^-test. In addition, interaction among variables was also evaluated in the logistic regression model. All statistical analyses were performed using InfoStat software (v2018).

### Spatial analysis

#### Descriptive analysis

The cartography was carried out by georeferencing the households of the volunteers included in the sampling; they were located on the map based on their geographical coordinates x and y. In turn, a digital elevation model of Tandil city was added, prepared from a 900 m^2^ satellite image from the SRTM (Shuttle Radar Topography Mission) mission. The resulting map was completed with information corresponding to the area reached by the running water and sewerage networks, each represented by a polygon over the city, making it possible to see what part of the population does not have these services.

### Cluster analysis

Potential spatial clusters were investigated in the study area with space scan statistics using SaTScan software, v10.0.2. A Bernoulli model for high rates and low rates was performed for detecting spatial patterns (22). Also, factors associated with the clusters of high and low rates of positivity were assessed through bivariate and multivariate analyses with InfoStat software (v2018).

Then, the distances of each address of the people included in the sampling, in relation to the nearest watercourse, were calculated using ArcGIS software v10.2. The thematic cartography was completed with two maps. The first shows the areas according to flood risk and the second shows the location of the four wastewater treatment plants in Tandil (23).

### Ethical statement

The study complied with the revised Declaration of Helsinki for biomedical research involving human subjects, and was approved by the Ethics Committee of the National Institute of Epidemiology “Dr. Juan H. Jara,” Mar del Plata, Argentina (Code: RIVERO -02/2020) and the Teaching and Research Committee of the Integrated Public Health System of Tandil. Prior to enrolment, the researchers read an information sheet describing the study, answered any questions, and asked for written consent to participate. The participants received no compensation for their participation and were free to withdraw from the study at any time. Anonymity was guaranteed using an identification code.

## Results

None of the participants withdrawed from the study. Overall, 45/969 serum samples (4.64%) tested were positive for anti-HEV IgG (CI 95% 3.27–6.02).

### Clinical characteristics related to the seropositivity

Although 5 out of 40 (13%) seropositive cases had a previous diagnosis of hepatitis, the differences were not significant compared to seronegatives (8/108, 7%) (Fisher *p* = 0.51). None of the seropositive cases was previously diagnosed as an HEV infection.

Table 1 shows the signs and symptoms of subjects at *p* < 0.2 in the bivariate analysis as well as the relation with the seropositivity to HEV infection.

**Table 1:** Clinical characteristics of participants from Tandil, Buenos Aires Province, Argentina.

Variables	Seropositives frequency (%)	Seronegatives frequency (%)	OR (CI95%)	*p* value
Dark urine	6/40 (15)	4/109 (4)	4.63 (1.31–16.36)	0.02*
Nausea and vomiting	7/41 (17)	9/107 (8)	2.24 (0.80–6.30)	0.14*
Chest pain	5/41 (12)	5/108 (5)	2.86 (0.83–9.89)	0.13*
Pulmonary disease	6/41 (15)	6/109 (6)	2.94 (0.93–9.31)	0.08*

Frequencies and association with HEV seropositivity. *Fisher exact test.

After the logistic regression analysis, the clinical manifestation associated with seropositivity to HEV was a history of dark urine OR 4.63 (CI 95% 1.23–17.39), *p* = 0.02.

### Risk factor analysis

Tables 2, 3 show the socio-demographic characteristics at *p* < 0.2 in the bivariate analysis as well as the relation with the seropositivity to HEV infection.

**Table 2:** Socio-demographic characteristics of participants from Tandil, Buenos Aires Province, Argentina.

Variables	Seropositives frequency (%)	Seronegatives frequency (%)	OR (CI95%)	*p* value
Socio-demographic variables				
Male gender	22/41 (54)	35/106 (33)	2.35 (1.13–4.86)	0.02
Age (years)				0.06*
0–10	1/41 (2)	2/107 (2)		
11–20	1/41 (2)	8/107 (7)		
21–30	2/41 (5)	16/107 (15)		
31–40	6/41 (15)	20/107 (19)		
41–50	9/41 (22)	30/107 (28)		
51–60	16/41 (39)	26/107 (24)		
61–70	2/41 (5)	4/107 (4)		
71–80	4/41 (10)	1/107 (1)		
Educational level of breadwinner: primary complete or more	30/39 (77)	89/102 (87)	0.49 (0.19–1.23)	0.13
Housing conditions				
Computer access at home	29/40 (73)	92/102 (90)	0.29 (0.11–0.73)	0.00
Public gas service	35/41 (85)	102/109 (94)	0.40 (0.13–1.22)	0.18*
Appropriate sewage disposal	37/41 (90)	104/107 (97)	0.27 (0.06–1.13)	0.09*
Near to natural water courses	11/41 (27)	16/108 (15)	2.11 (0.89–4.97)	0.08
Near to livestock productions	3/41 (7)	2/108 (2)	4.18 (0.79–22.10)	0.12*
Presence of rodents inside the house	7/40 (18)	6/105 (6)	3.5 (1.14–10.74)	0.04*

Frequencies and association with HEV seropositivity. *Fisher exact test.

**Table 3:** Quantitative socio-demographic characteristics variables of respondents from Tandil, Buenos Aires Province, Argentina and association with HEV seropositivity.

Variable	Seropositives median (Q1–Q3)	Seronegatives median (Q1–Q3)	Wilcoxon test *p* value
Age (years)	51 (42–59)	42 (31–52)	0.00

In Tables 4, 5, occupations and other exposure activities of respondents at *p* < 0.2 in the bivariate analysis as well as the relation with the seropositivity to HEV infection are shown.

**Table 4:** Occupations and other exposure activities of participants from Tandil, Buenos Aires Province, Argentina.

Variables	Seropositives frequency (%)	Seronegatives frequency (%)	OR (CI95%)	*p* value
Occupation or exposure activities				
Caregiver	9/40 (23)	14/108 (13)	1.95 (0.78–4.85)	0.15
Rural worker	12/41 (29)	9/107 (8)	4.51 (1.76–11.52)	0.00
Assist an animal giving birth	5/41 (12)	4/108 (4)	3.61 (0.98–13.27)	0.06*
Pruner	13/40 (33)	23/108 (21)	1.78 (0.8–3.94)	0.15
Temporary worker	2/41 (5)	1/108 (1)	5.49 (0.70–42.92)	0.18*
Slaughterer	2/41 (5)	1/108 (1)	5.49 (0.70–42.92)	0.18*
Individual exposures				
Water sports practice	24/39 (62)	47/104 (45)	1.94 (0.92–4.08)	0.08
Exposure to river water	15/35 (43)	22/99 (22)	2.63 (1.17–5.90)	0.01
Exposure to wastewater	11/35 (31)	16/101 (16)	2.43 (1.01–5.85)	0.04
Handwashing	38/41 (93)	106/106 (100)	–	0.02*
History of travel to Europe	12/37 (32)	17/93 (18)	2.15 (0.91–5.04)	0.08
Food consumption history				
Homemade sausage	21/41 (51)	42/109 (39)	1.68 (0.82–3.43)	0.16
Consuming undercooked frankfurter	3/41 (7)	1/108 (1)	8.45 (1.2–59.25)	0.06*
Consuming undercooked bovine meat	10/41 (24)	14/109 (13)	2.19 (0.90–5.33)	0.08
Other individual exposures				
Contact with a person with hepatitis	17/40 (43)	25/103 (25)	2.31 (1.07–4.95)	0.03
Visiting, living or working in the rural area	26/39 (67)	43/108 (40)	3.02 (1.41–6.46)	0.00
Contact with animals				
Horses	13/41 (32)	19/109 (17)	2.20 (0.98–4.95)	0.05
Sheep	12/41 (29)	18/108 (17)	2.07 (0.90–4.74)	0.08
Deer	4/41 (10)	2/107 (2)	5.68 (1.16–27.84)	0.05*

Frequencies and association with HEV seropositivity. *Fisher exact test.

**Table 5:** Quantitative occupations and other exposure activities of respondents from Tandil, Buenos Aires Province, Argentina and association with HEV seropositivity.

Variable	Seropositives median (Q1–Q3)	Seronegatives median (Q1–Q3)	Wilcoxon test *p* value
Distance (m) to water courses	1354.62 (779.16–1985.81)	1562.42 (938.61–1805.29)	0.03

After the logistic regression analysis, the significant predictors that best explained seropositivity to HEV were the presence of natural water courses near the households (OR: 3.24, 95% CI: 1.16–9.07, *p* = 0.02); the age as a quantitative variable (OR: 1.03, 95% CI: 1.00–1.06, p 0.04); and a history of previous travel to Europe (OR: 2.66, 95% CI: 1.01–7.01, *p* = 0.04) (Table 6).

**Table 6:** HEV seropositivity predictors in individuals from Tandil, Buenos Aires Province, Argentina as determined by multivariate logistic regression model.

Parameters	Est	SE	OR	Wald LI (95%)	Wald LS (95%)	Wald Chi^2^	*p* value
Intercept	2.94	0.77	0.05	0.01	0.24	14.42	0.00
Previous travel to Europe	0.98	0.49	2.66	1.01	7.01	3.93	0.04
Natural water courses near the households	1.18	0.52	3.24	1.16	9.07	5.02	0.02
Presence of rodents inside the house	1.18	0.66	3.27	0.90	11.81	3.26	0.07
Age (years)	0.03	0.02	1.03	1.00	1.06	4.18	0.04

### Spatial analysis

**Figure 2:**
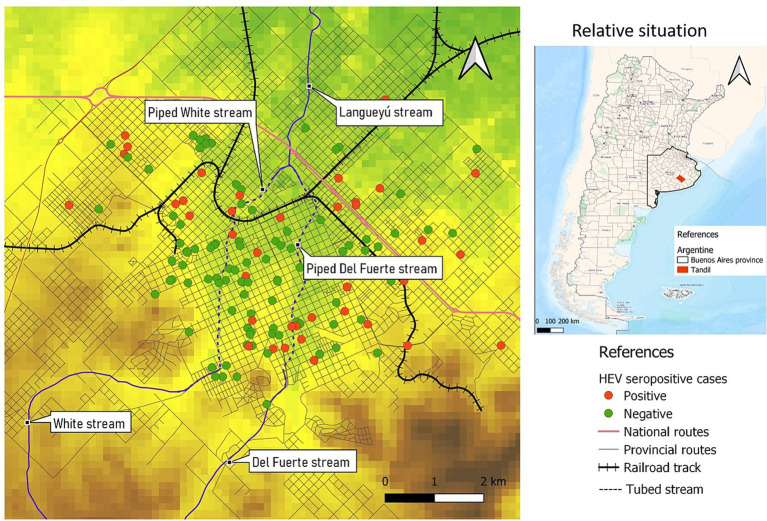
Spatial distribution of HEV seropositive cases and controls, Tandil, Buenos Aires Province, Argentina.

The spatial distribution of the seropositive and seronegative cases is shown in Figure 2.

**Figure 3:**
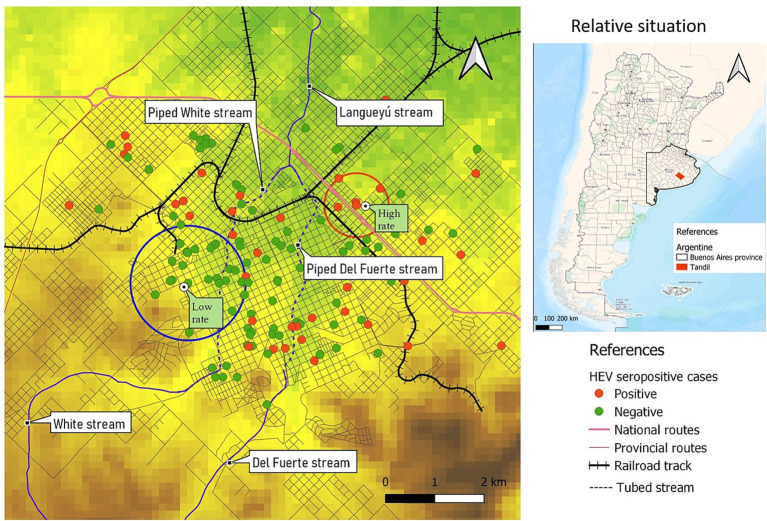
Spatial clusters of higher and lower risk of HEV.

A spatial cluster of high rates of HEV seropositive cases was detected (coordinates of centroid: 37.308172S, 59.115827W; radius: 0.60 km). The entire population within the cluster, consisting of 5 individuals, exhibited seropositivity with a prevalence rate of 100% (*p* = 0.08). Additionally, a spatial cluster characterized by a low rate of HEV seropositivity cases was identified. The centroid of this second cluster was located at coordinates 37.323207S, 59.154066W, with a radius of 1.16 km. Within this cluster, a population of 25 individuals was observed, and none of them tested positive for HEV (0% seropositivity, *p* = 0.01) (Figure 3).

**Figure 4:**
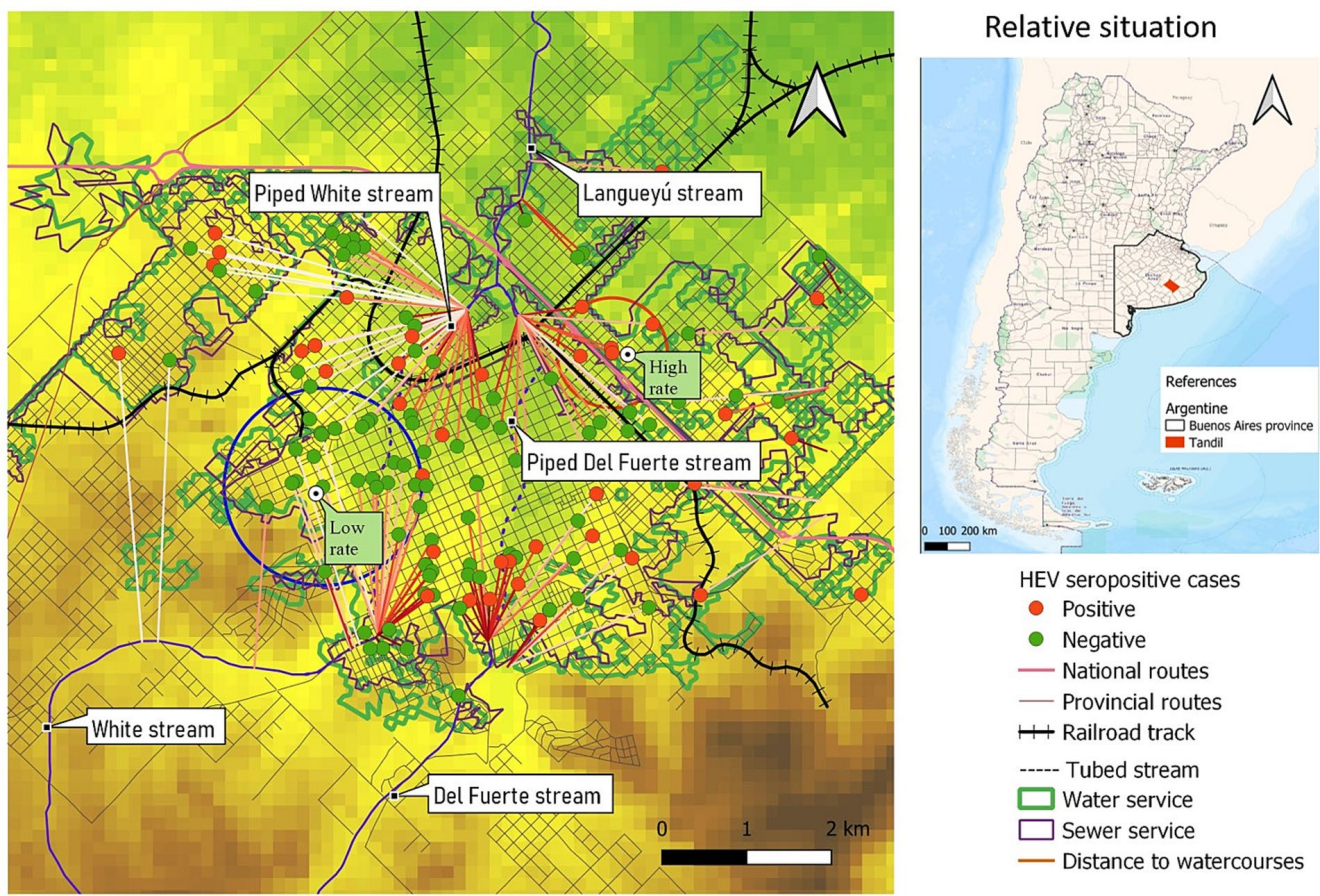
Distance to the nearest watercourses. Area covered by water and sewage services.

Most of the individuals have access to appropriate sewage disposal and to safe drinking water at home (Figure 4). Significant differences in the proximity to water courses can be observed between individuals residing within the high-rate spatial cluster and those within the low-rate spatial cluster. Specifically, individuals living within the high-rate cluster tend to be in closer proximity to water courses compared to individuals residing within the low-rate cluster.

**Figure 5:**
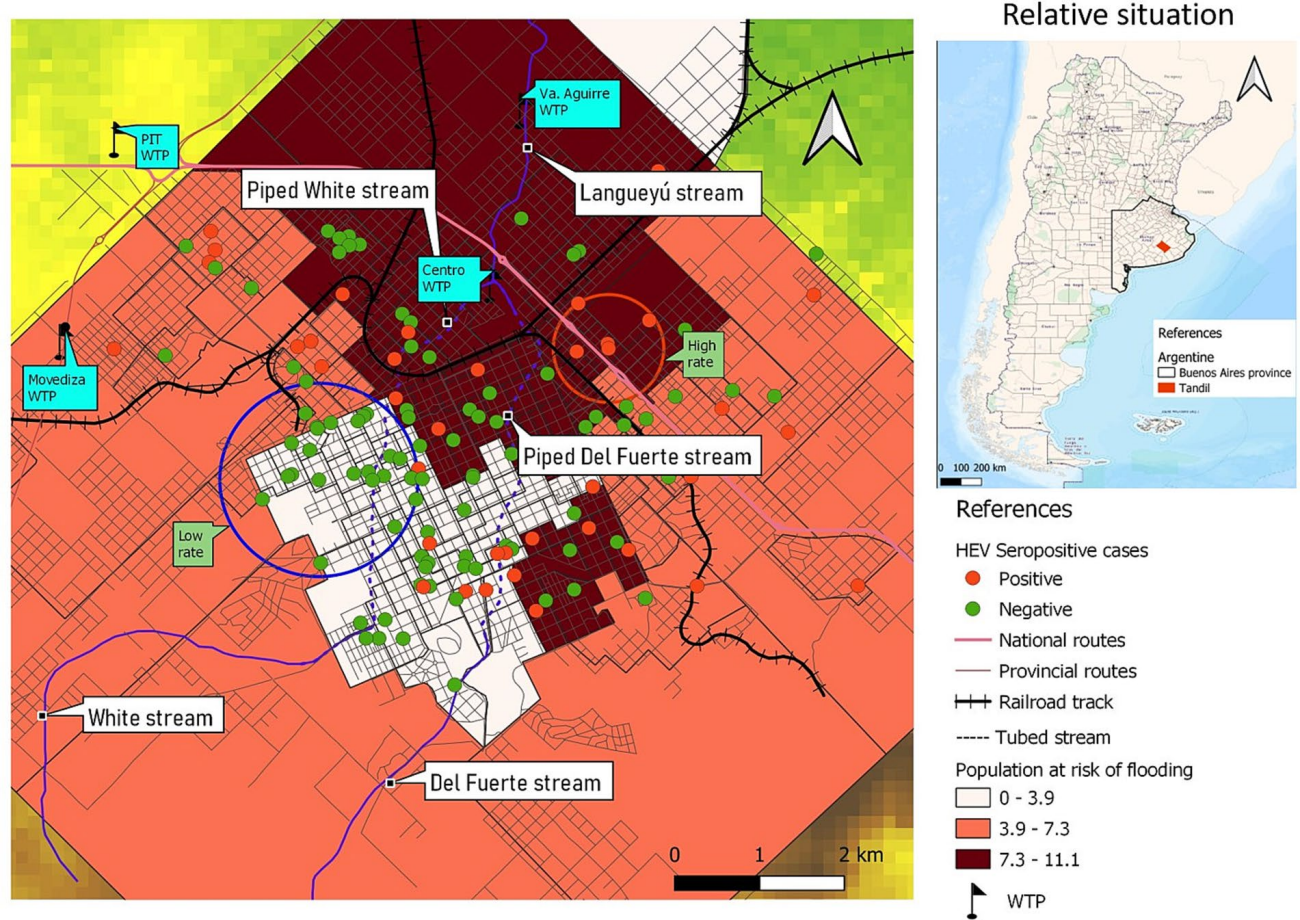
Population at risk of flooding and location of water treatment plants (WTP) in Tandil city.

The high-risk spatial area is not only associated with increased risk but also coincides with regions prone to flooding during precipitation events. Additionally, it is situated in close proximity to the Langueyú stream (Figures 1, 4, 5). Furthermore, near the high-risk spatial cluster, there is a wastewater treatment plant (WTP) situated. Conversely, the occurrence of flooding events in the low-risk region is uncommon, as depicted in Figure 5.

In the evaluation of factors associated with residing within the high-rate spatial cluster, several variables showed significance (*p* < 0.2) in the bivariate analysis. These variables, as presented in Table 7, include male sex, engagement in rural work, and the proximity of water courses to residential areas.

**Table 7:** Variables at *p* < 0.2 related with being inside the high-risk spatial cluster of HEV seropositivity.

Variables	Inside the high-risk cluster frequency (%)	Outside the high-risk cluster frequency (%)	OR (CI95%)	*p* value
Male gender	4/5 (80)	53/142 (37)	6.72 (0.73–61.69)	0.07*
Rural work	2/5 (40)	19/143 (13)	4.35 (0.68–27.75)	0.14*
Household near to natural water courses	3/5 (60)	24/144 (17)	7.5 (1.18–47.32)	0.04*

Tandil, Buenos Aires Province, Argentina. *Fisher exact test.

After the logistic regression analysis, the significant predictor that best explained being inside the high-risk spatial cluster of seropositivity to HEV was the presence of natural water courses near the households (OR: 7.50, 95% CI: 1.19–47.32, *p* = 0.03).

When assessing for factors associated with living inside the low-rate spatial cluster, variables at *p* < 0.2 in the bivariate analysis were: Male sex, rural work, distance to water courses and previous travel to Europe. All of them were inversely related to the low-risk cluster (Tables 8, 9).

**Table 8:** Variables at *p* < 0.2 related with being inside the low-risk spatial cluster of HEV seropositivity.

Variables	Inside the low-risk cluster frequency (%)	Outside the low-risk cluster frequency (%)	OR (CI95%)	*p* value
Male gender	3/25 (12)	54/122 (44)	0.17 (0.05–0.60)	0.00
Rural work	0/25 (0)	21/123 (17)	–	0.02*
Travel to Europe	0/22 (0)	29/108 (27)	–	0.00*

Tandil, Buenos Aires Province, Argentina. *Fisher exact test.

**Table 9:** Quantitative variable related with being inside the low-rate spatial cluster of HEV seropositivity.

Variable	Individuals inside the low rate spatial cluster median (Q1–Q3)	Individuals outside the low rate spatial cluster median (Q1–Q3)	Wilcoxon test *p* value
Distance (m) of the household to water courses	1736.46 (1661.91–1930.14)	1323.36 (818.64–1788.95)	0.00

Tandil, Buenos Aires Province, Argentina.

After the logistic regression analysis, the significant predictors that best explained being inside the low-risk spatial cluster of seropositivity to HEV were the distance of natural water courses (OR 1.08; 95% CI 1.02–1.16; *p* = 0.01) and the male sex (OR 0.17; 95% CI 0.05–0.60; *p* = 0.00). Previous travel to Europe and rural activities was not incorporated into logistics because there were no individuals in the cluster with this characteristic.

## Discussion

### Seroprevalence

Overall, 4.65 (95% CI 3.27–6.02) of the sera were positive. Although comparisons between studies are difficult due to differences in the demographics of the population studied and in the HEV antibody detection assays used, the results are similar to the levels reported in the Americas, that varies from 0 to 40.6%, as reported by Fernandez Villalobos et al. (2). And it is also close to the 5.6% seroprevalence seen in an indigenous population in northern Argentina, slightly higher to that reported in Córdoba (3.47%) and in Salta (9%), but lower to the seroprevalence observed in Tucumán (9.23%), all cities from Argentina (14–17).

In contrast to the aforementioned countries and regions where higher seroprevalence rates were reported (South Africa: 42.8%, Thailand: 29.7%, Bulgaria: 25.9%, Croatia: 21.5%, India: 17.7%, Serbia: 15%, southwest France: 16.6%, southwest England: 15.8%, Denmark in 2008: 20.6%, and the USA in 2002: 18.3%), the seroprevalence rate observed in Tandil is significantly lower (5, 6, 24). In the previously mentioned studies, seroprevalences correspond to sera from blood donors and not from the general population, differing from this study in the age of the individuals included.

### Clinical signs and symptoms

None of the 41 seropositive cases had a previous diagnosis of HEV infection. Lack of knowledge among physicians and an absence of standardized diagnostic tests may result in increased morbidity and mortality from HEV infection (25).

Previous history of dark urine remained statistically significant after the logistic regression analysis. This sign was described before, related with the icteric phase of clinical presentation of acute hepatitis that may be prolonged for weeks or months in some cases. It should be considered for differential diagnosis, since hepatitis E seropositive cases presented this sign 4.63 times more often than seronegatives (7).

### Risk factor and spatial analysis

#### Socio-demographic variables

Despite not showing association in the logistic regression analysis, it is noteworthy that seropositive cases exhibited limited access to computers at home (*p* < 0.05). This variable, serving as an indirect indicator of socioeconomic status, indicates that the prevalence of HEV was higher among individuals from low income households. The reduced access to computers in poorer homes suggests a correlation between lower socioeconomic status and higher prevalence of HEV as it was reported in other regions of Latin America (26).

In the univariate analysis, male sex was found to be statistically associated with HEV infection and living inside the low-risk spatial cluster was inversely related with male sex; this result coincides with previous reports (27, 28). The higher incidence of the disease among males has been attributed to an increased presence of behavioral risk factors compared to females. Furthermore, men often engage in various environmentally related tasks that are traditionally considered “men’s jobs.” These tasks include activities such as irrigation farming using contaminated river water, and disposal of human and animal waste, swine veterinarians, pig slaughterers, meat inspectors, and sewage - workers.

Similar to other studies (2, 26, 30), we found that an increasing age is associated with HEV seroprevalence, mainly due to cumulative exposure but it may also be related to different lifestyles of the older adult.

### Water exposure

Presence of natural water courses near the households was the exposure variable most related with a previous history of HEV infection. HEV cases were found to reside near water courses 3.24 times more often compared to seronegative cases. Additionally, the bivariate analysis revealed a significant association between exposure to river water and wastewater.

A spatial region with a high rate of cases was determined, in a peri-urban area that is close to agricultural areas and livestock. Moreover, people living inside the high-risk spatial cluster were 7.5 times closer to water courses than people living outside this area. Besides, the high-risk spatial cluster not only coincides with an area that is susceptible to flooding during precipitation events but is also situated in close proximity to the Langueyú stream. Also, a sewage treatment plant was located near the high-risk spatial cluster. It is noteworthy that the operation of the plant is altered by clandestine connections of home storm drains to the sewerage network. Furthermore, the stormwater drainage network, which includes piped streams, is affected by clandestine connections of industrial and sewage effluents (18). On the opposite, in the low-risk spatial cluster flooding events are unusual and people live more distant to water courses.

Contaminated water exposure is believed to have a significant impact on the transmission of various HEV subtypes, particularly in cases where direct zoonotic exposure is not involved. Several studies have highlighted the potential risk of environmental contamination in watersheds (such as rivers and dams) and the water distribution network due to the discharge of untreated urban wastewater or wastewater from pig slaughterhouses. This contamination can lead to waterborne infections among the exposed population. The notably high frequency of HEV RNA in urban sewage samples from Spain, the US, France and Israel clearly highlights the environmental presence of HEV (3, 31). This fact is underlined by the detection of HEV RNA in various water sources, especially during outbreaks of hepatitis E. Subclinical and sporadic infection in humans turns them into HEV reservoirs, being able to contaminate the environment through their feces (5). In turn, animals such as pigs or wild animals can act as reservoirs and can also directly impact the soil or surface waters, for example, by bathers or by defecation (32).

Today, most urban areas in the developing world still lack sufficient sewage treatment infrastructure. This deficiency not only results in significant ecological degradation of their waters but also poses substantial risks to human health. In unsewered urban areas, overflow from septic tanks and drainage from cesspools may enter surface waters via groundwater, and this pollution will largely act separately from the effects of urban stormwater runoff. Better knowledge on the source of HEV contamination, occurrence, persistance in water, and removal by water treatment is needed to unravel this transmission path (18, 32).

### Animal contact

While HEV has the capability to infect various species such as bats, ferrets, rabbits, and chickens, the main reservoirs responsible for transmitting the virus to humans are swine, deer, and wild boar. Among these species, swine are widely regarded as the primary reservoir of HEV infection (25). There is a need to compile evidence on the zoonotic dissemination of the virus in animal hosts and the environment. In this work, the bivariate analysis revealed that seropositive cases presented rodents inside their houses with more frequency than seronegatives, although this was not statistically significant after logistic regression analysis. Besides, being a rural worker as well as visiting, living, or working in the rural area were related with the infection in the bivariate. Moreover, living inside the low-risk spatial cluster was inversely related to rural work. These exposures are also associated with contact with domestic animals.

Additionally, contact with deer was particularly identified as a risk factor in the bivariate analysis. This result coincides with (33) reports, who provided evidence of zoonotic transmission of HEV infection from deer to humans. Also, the presence of HEV RNA or antibodies has been described in deer, swine, cows, goats, and rodents, but no data is available for the area where this study was performed (34).

### Other individual exposures

Prevention can be achieved through the provision of good basic hygiene. Handwashing was found to be a protective factor in the bivariate analysis. Moreover, in this study, previous contact with a case of hepatitis was associated with seropositivity in the bivariate analysis.

Previous travel to Europe was also related with seropositivity cases. Moreover, people living inside the low-risk spatial cluster were inversely associated with being in Europe. According to data published by European Centre for Disease Prevention and Control, the number of confirmed HEV cases in the European Union (EU) has been increasing each year from 514 in 2005 to 5,617 cases in 2015, representing a 10-fold increase. The most common way to become infected with HEV in the EU is through the consumption of raw or undercooked pork meat and liver (ECDC Report, 2017) (35). Furthermore, evidence suggests that HEV is an under-recognized pathogen in high-income countries. The actual number of human infections due to HEV in Europe is still unclear, given the widespread variations in clinical awareness, and testing surveillance practices, and a general lack of published information across the majority of EU/EEA Member States (35).

Consumption of undercooked HEV-contaminated food, such as meat, milk (cow, goat, sheep, and donkey) and molluscs, have been related to the infection; in this study, seropositive individuals had consumed more frequently homemade sausage, undercooked frankfurter and undercooked bovine meat than seronegative, although the differences were not statistically significant (36).

## Conclusion

HEV seroprevalence estimated among the general population of Tandil city was 4.34%. Different factors related to the infection and a spatial pattern of high and low risk were determined, showing HEV multiple means of transmission. It is necessary to promote specific preventive actions (proper personal hygiene, handwashing after contact with animals, correct cook of animal products, provision for adequate clean drinking water, good environmental sanitation, proper disposal for animal and human feces) and specific diagnosis of HEV in the region under study, considering the populations with the highest risk of infection (people living near water courses and in floodable regions, adult men, rural worker, people traveling to Europe, etc.). Additionally, it is necessary to consider HEV in the differential diagnosis in individuals presenting acute hepatitis, particularly in populations susceptible to developing severe diseases, such as pregnant women, patients with chronic liver disease and immunocompromised patients. Considering it is an endemic disease in the region, it would be necessary to include HEV in the blood screening policy at donation centers to avoid transmission through blood transfusion, particularly to those who present a higher risk of experiencing more severe consequences.

These considerations could also be applied in other regions with similar socioeconomic characteristics.

## Data availability statement

The raw data supporting the conclusions of this article will be made available by the authors, without undue reservation.

## Ethics statement

The studies involving humans were approved by National Institute of Epidemiology “Dr. Juan H. Jara,” Mar del Plata, Argentina. The studies were conducted in accordance with the local legislation and institutional requirements. Written informed consent for participation in this study was provided by the participants’ legal guardians/next of kin.

## Author contributions

MR: Conceptualization, Data curation, Formal analysis, Funding acquisition, Investigation, Methodology, Project administration, Resources, Supervision, Visualization, Writing – original draft, Writing – review & editing. LA: Investigation, Resources, Validation, Writing – review & editing. SG: Conceptualization, Investigation, Resources, Writing – review & editing. AT: Data curation, Funding acquisition, Methodology, Resources, Visualization, Writing – review & editing. JP: Data curation, Visualization, Writing – review & editing. JS: –. AB: Investigation, Writing – review & editing. FS: Investigation, Writing – review & editing. JM: Investigation, Writing – review & editing. SE: Conceptualization, Investigation, Writing – review & editing. MV-P: Funding acquisition, Project administration, Resources, Supervision, Validation, Writing – review & editing.
