# Supplementary material for: Exploring hepatitis E virus seroprevalence and associated risk factors among the human population in Tandil, Buenos Aires, Argentina
Source: Front Public Health. 2023 Oct 5;11:1257754. doi: 10.3389/fpubh.2023.1257754 (PMC10585172; doi:10.3389/fpubh.2023.1257754)
Supplement: Supplementary file 1 [file Data_Sheet_1.pdf]

ENCUESTA PROYECTO DE HEPATITIS E

Código: ____________ Fecha de TOMA DE MUESTRA: / / Encuestador:

1. DATOS PERSONALES

Nombre y Apellido: **Teléfono:**

DNI: **Fecha de nacimiento: / / Edad: Nacionalidad:**

Sexo: Hombre □ Mujer □ Se desconoce □

Número de teléfono (móvil): **Número de teléfono (fijo):**

Dirección (calle y número): **Latitud/ Longitud:**

2. *DATOS SOCIOECONÓMICOS*

Cobertura de salud (Obra Social/Prepaga): Si □ No □

Máximo nivel educativo del principal sostén del hogar: secundario completo o más Si □ No □

Cantidad de individuos ocupados en el hogar:

Usa notebook, netbook, Tablet, PC de escritorio, etc. (el encuestado): Si □ No □

En la vivienda algún niño en edad escolar no asiste a la escuela: Si □ No □

NBI: Si □ No □

3. CARACTERÍSTICAS DE LA VIVIENDA

3.1 **Número de personas que residen en el hogar**: **Total de habitaciones para dormir:**

3.2 Hacinamiento (más de 2 habitantes por cuarto): Si □ No □

3.3 Viviendas precarias: incluye casillas, piezas en inquilinato, locales no construidos para habitación y viviendas móviles (no se consideran los hoteles y pensiones): Si □ No □

3.4 Posee baño/letrina: Si □ No □ 3.5 Desagüe del inodoro a red pública (cloacas): Si □ No □

3.6 Agua procedente de red pública (agua corriente): Si □ No □ 3.7 Gas de red: Si □ No □

3.8. El techo de la vivienda es de:

Tejas □ Chapas □ Losa □ Otros □ (especificar)

3.9. **El piso de la vivienda es de**

Madera □ Cemento □ Baldosa/cerámico □ Tierra □ Otros □ (especificar)

3.10. Las paredes de la vivienda son de

Madera □ Cemento □ Ladrillos □ Chapas □ Barro/Adobe □ Otros □ (Especificar)

3.11. ¿Hay roedores en la vivienda? No □ Si □

3.12. ¿Hay roedores en la cercanía de la vivienda? No □ Si □

3.13. En la proximidad (menos de 100 metros) de la casa hay

	SI	NO		SI	NO
Arroyos, ríos, desagües, lagunas, pantanos o zanjones			Calles inundables		
Baldíos			Corrales (gallinero/chanchería/etc.)		
Basurales			Explotaciones pecuarias		

4. ANTECEDENTES EPIDEMIOLÓGICOS PERSONALES

4.1 ¿Tiene o tuvo contacto con animales? *Tipo de Contacto (Ocupacional/ Hogar/ Recreacional/ Autoconsumo/ Otro)

	SI	NO	Tipo de contacto*		SI	NO	Tipo de contacto*
Porcinos				Felinos			
Jabalíes				Ovinos			
Bovinos				Ciervos			
Caninos				Aves de corral			
Equinos				Otros			

Otros: ¿CUALES?

4.2 ¿Accede o visitó la zona rural? No □ Si □

4.3 ¿Realiza o realizó alguna de las siguientes actividades? Marcar con una cruz lo que corresponda

	SI	NO		SI	NO
Veterinario/Ayudante			Changarín		
Tareas rurales			Limpieza/ poda de ramas		
Asistencia de partos en animales			Militar		
Laboratorista			Obrero de la construcción/excavador		
Aplicación vacuna brucelosis			Pescador		
Caza de animales silvestres			Plomero/ gasista		
Estudiante Medicina/Veterinaria			Quintero		
Elaboración de embutidos caseros			Recolector de residuos		
Elaboración de lácteos caseros			Trabaja en canteras		
Matarife/Empleado de frigorífico			Cocinero		
Profesional de la salud			Transporte de bovinos		

Biólogo			Transporte de porcinos		
Carnicero			Venta de productos de origen porcino		
Cartonero			Venta de productos de origen bovino		
Cuidado de personas (niños, ancianos)			Otros (especificar)		

Si la respuesta fue sí a alguna de las anteriores:

Para su protección utiliza*:*

Guantes No □ Si □ Botas No □ Si □ Barbijos No □ Si □ Otros No □ Si□

***4.4.* Deportes o actividades recreativas de contacto con agua: No □ Si□**

Tipo de agua: Lago/Laguna No □ Si □ Río/Arroyo No □ Si □ Mar No □ Si □

Otros No □ Si □ Especifique:

***4.5* ¿Tuvo contacto con aguas no seguras? No □ Si □**

Tipo de agua: Lago/Laguna□ Arroyo □ Zona inundada□ Aguas residuales (pozos ciegos/cloacas, etc) □

4.6. Antecedentes de viajes

-Asia (Medio Oriente/India) *No□ Si□ ¿A qué país?* **Fecha: / /**
-Norte de África *No □ Si □ ¿A qué país?* **Fecha: / /**
-Europa *No □ Si □ ¿A qué país?* **Fecha: / /**
-América Central *No □ Si □ ¿A qué país?* **Fecha: / /**
-América del Norte *No □ Si □ ¿A qué país?* **Fecha: / /**
-América del Sur No □ Sí □ *¿A qué país?* **Fecha: / /**
-Otro lugar *No □ Si □ ¿A qué país?* **Fecha: / /**

4.7 Antecedentes de consumo

	SI	NO		SI	NO
Productos lácteos sin pasteurizar			Mariscos insuficientemente cocidos		
Embutidos de cerdo caseros			Pescado insuficientemente cocido		
Carne de vaca insuficientemente cocida			Carne de cerdo insuficientemente cocida		
Fruta sin lavar			Carne de jabalí insuficientemente cocida		
Verdura cruda sin lavar			Carne de ciervo insuficientemente cocida		
Agua de pozo sin hervir			Salchichas comerciales insuficientemente cocidas		
Agua de lluvia			Embutidos de jabalí caseros		

Consume bebidas con alcohol No □ Si □

¿Con qué frecuencia? 4 o más veces por semana □ 2-3 veces por semana□ 2 a 4 veces al mes□ 1 vez al mes o menos□ Nunca□

¿Cuántas bebidas* que contienen alcohol toma en un día típico cuando bebe? 0 - 2 □ 3 o 4 □ 5 o 6 □ 7 - 9 □ 10 o más □

***Una bebida equivale a: 1 lata de cerveza o media copa de vino o un trago.**

4.8. Otros antecedentes previos:

Antecedentes de transfusión No □ Si □ Antecedentes de transplante de órganos No □ Si □

Contacto con algún caso de hepatitis No □ Si □ Antecedente de hepatitis en el embarazo de la madre No □ Si □

4.9. ¿Se lava siempre las manos al volver a su casa? No□ Si□

5. COMORBILIDADES

Peso: Altura: IMC: Grupo Sanguíneo:

HTA Si □ No □ Diabetes Si □ No □ Insuficiencia cardíaca Si □ No □

Enfermedades reumatológicas Si □ No □ Ex fumador/Fumador Si □ No □

Enfermedad oncológica Si □ No □ ¿cuál?

6. SIGNOS Y SÍNTOMAS

¿Tuvo o le diagnosticaron?

	**SI**	**NO**	**<6 meses**	**>6 meses**		**SI**	**NO**	**<6 meses**	**>6 meses**
Brucelosis					Falla hepática				
Dolor articular					Hemorragias (sangrados)				
Dolor de cabeza					Síndrome meníngeo				
Pérdida del apetito					Dolor abdominal				
Dolor muscular					Prurito (picazón)				
Fiebre					Orina oscura				
Dificultad para dormir					Heces pálidas				
Sudoración extrema					Hepatitis				
Dolor en la espalda					Parásitos en materia fecal				

Cansancio					Cirrosis				
Malestar general					Falla renal				
Náuseas/Vómitos					Síntomas gripales				
Diarrea					Leptospirosis				
Dolor en el pecho					Varicela				
Tos					Coloración amarillenta de la piel y las mucosas				
Depresión					Pérdida de peso				
Estreñimiento					Alguna enfermedad exantemática (con ampollas, pústulas, vesículas) sin diagnóstico				
Impotencia sexual (sólo en hombres)					Herpes Zoster o Culebrilla				
Nerviosismo					Enfermedad renal				
Esplenomegalia					Ojos rojos				
Hepatomegalia					Varicela				
Artritis					Endocarditis				
Espondilitis					Fatiga crónica				
Epidídimo-orquitis					Congestión nasal				
Prostatitis					Enfermedad pulmonar				
Encefalitis					Toxoplasmosis				
Meningitis					Abortos				

Otros signos y síntomas (especificar):

7. Conocimiento sobre Hepatitis E

7.1 ¿Había recibido información? No □ Si □

7.2 En caso afirmativo quién se la brindó

Profesional de la Salud No □ Si □ Internet No □ Si □ Escuela No □ Si □ Medios de comunicación No □ Si □ Laboral No □ Si □ Otros No □ Si □ Especificar:

7.3 ¿Puede describir la enfermedad? No□ Si□ Describa

7.4. ¿Conoce medidas de prevención? No□ Si□ Especifique
